# A Study into the Evolutionary Divergence of the Core Promoter Elements of *PRPF31* and *TFPT*

**DOI:** 10.4172/1747-0862.1000067

**Published:** 2013-08

**Authors:** Anna M. Rose, Amna Z. Shah, Giovanna Alfano, Kinga M. Bujakowska, Amy F. Barker, J Louis Robertson, Sufia Rahman, Lourdes Valdés Sánchez, Francisco J. Diaz-Corrales, Christina F. Chakarova, Abhay Krishna, Shomi S. Bhattacharya

**Affiliations:** 1 Department of Genetics, UCL Institute of Ophthalmology, London, United Kingdom; 2 Ocular Genomics Institute & Berman-Gund Laboratory, Department of Ophthalmology, Harvard Medical School, Boston, USA; 3 Andalusian Molecular Biology and Regenerative Medicine Centre (CABIMER), Seville, 41092, Spain

**Keywords:** Photoreceptor, Dual-luciferase reporter, *PRPF31* mutation, *TFPT*

## Abstract

Mutations in *PRPF31* have been implicated in retinitis pigmentosa, a blinding disease caused by degeneration of rod photoreceptors. The disease mechanism in the majority of cases is haploinsufficiency. Crucially, attempts at generation of animal models of disease have proved unsuccessful, yielding animals with a visual phenotype that does not mirror human disease. This suggests that, in these animals, the transcriptional regulation of *PRPF31* is different to humans and compared to other species. Study of the evolution of the *PRPF31* core promoter has important implications for our understanding of human disease, as disease phenotype is modified by differentially expressed alleles in the population.

*PRPF31* lies in a head-to-head arrangement with *TFPT*, a gene involved in cellular apoptosis. The two genes were shown to share common regulatory elements in the human genome. In this study, the core promoters of *PRPF31* and *TFPT* were characterised by dual-luciferase reporter assay using genomic DNA from the green monkey, domestic dog and house mouse. It was found that the core promoters were conserved between human and monkey.

In dog, the *TFPT* core promoter was conserved, but different *PRPF31* gene architecture meant the gene was controlled by a long-range promoter lying some 2000bp from the transcription start site.

There was very low level of conservation (<20%) of the *PRPF31* 5′ region between mouse and human. It was shown that mouse populations did not show variable *Prpf31* expression levels, revealing a potential explanation for the lack of phenotype observed in the *Prpf31* knock-out mouse model.

## Introduction

*PRPF31* encodes the ubiquitous splicing factor PRPF31, an essential component of the U4/U6.U5 tri-snRNP. The gene is highly conserved throughout evolution, with orthologues in all vertebrate species, invertebrates and lower species, including yeast. Mutations in *PRPF31* have been shown to be a major cause of autosomal dominant retinitis pigmentosa (adRP), accounting for 5% of disease in the UK [1,2].

A unique feature of *PRPF31*-associated adRP is phenotypic non-penetrance, where within affected families there are asymptomatic mutation carriers. This is due to the existence of differentially expressed wildtype *PRPF31* alleles, with co-inheritance of a *PRPF31* mutation and a higher-expressing allele providing protection against clinical manifestation of disease. It has been shown that there is variable expression of *PRPF31* in the general population and, that within mutation carrying families, asymptomatic mutation carriers have more than two-fold higher expression levels of wildtype *PRPF31* compared to symptomatic individuals [3–6].

One study looked at phenotypic discordance between mutation-carrying siblings and observed that the symptomatic and asymptomatic siblings consistently inherited different wildtype chromosome 19q13 alleles from the non-mutation carrying parent [7]. It is generally thought, therefore, that *cis-*acting factors that affect the level of *PRPF31* expression (such as regulatory region polymorphisms) underlie phenotypic non-penetrance in mutation-carrying families. However, attempts to identify such changes have not yet been successful.

It has also been demonstrated that there is increased expression of both *PRPF31* alleles in asymptomatic mutation-carrying individuals, with subsequent degradation of the mutant molecule by nonsense mediated decay – this indicating that at least one factor that alters *PRPF31* expression acts in *trans* [8]. One possible *trans*-acting factor was identified through the association of higher *PRPF31* expression and an expression quantitative trait locus (eQTL) at 14q21-23, although the exact factor was not characterized [3]. It was also shown that variable expression of *CNOT3* is an important factor in determining *PRFP31* expression level - with increased levels of CNOT3 protein causing transcriptional repression of *PRPF31* [9]. CNOT3 is a component of the Ccr4-Not transcription complex, which is a global regulator of RNA polymerase II-mediated transcription [10].

Attempts to generate mouse models of *PRPF31*-associated adRP have failed to yield animals with a retinal degeneration phenotype. Neither *Prpf31* knock-in animals nor knock-out animals displayed retinal degeneration, and the animals did not have any visual defect at up to 18 months of age [11]. There is some evidence that *Prpf31* knockout mice develop changes within the retinal pigment epithelium (RPE), with vacuolation, loss of the basal infoldings and accumulation of amorphous deposits between the RPE and Bruch’s membrane [12]. There was not, however, death of retinal photoreceptor cells (the primary histological and pathological change in human disease) and no change in retinal function was reported – and so these animals cannot be considered a model for human disease [12]. It is possible that the same RPE changes are observed in asymptomatic individuals, but this study is not feasible. It can be concluded that in mouse, 50% of protein level is sufficient for normal retinal function.

Regulation of gene expression is central to pathogenesis of *PRPF31* mutations in humans and also the failure of animal models of disease and it is necessary, therefore, to understand the 5′ architecture of the *PRPF31* gene. *PRPF31* and *TFPT* are arranged in a bidirectional gene pair, with partially shared exon 1, at chromosome 19q13.4. It is increasingly recognised that many genes exist in bidirectional pairs, which are defined as two genes that lie in a head-to-head arrangement, on opposite DNA strands, with less than 1kb separating their transcription start site (TSS).

*TFPT*, also known as *CF3 fusion partner* or *FB1*, encodes a 253 amino acid protein, that was first identified in some cases of paediatric pre-B-cell acute lymphoblastic leukaemia as the fusion partner of the transcription factor E2A [13]. A role for the human protein has not yet been described, but the rat homologue, *Tfpt*, has been shown to be pro-apoptotic and might modulate cerebral apoptosis [14].

As differential expression of *PRPF31* underlies phenotypic non-penetrance, a study was undertaken to characterize the core promoter element of the gene (and the bidirectional gene pair, *TFPT*), as it was considered important to understand the transcriptional regulation of the gene in the normal population [15]. The work by Rose et al. [15] repeated and extended a previous promoter characterization study by Brambillasca et al. [16], which had assayed fragments from the reverse strand in order to characterize a putative *TFPT* promoter element. Dual luciferase reporter assay was performed and a fragment termed BiP was defined as the core promoter of *PRPF31*, whereas the core promoter of *TFPT* was defined as a fragment termed P.31-Luc [15].

It was considered that studying the conservation and evolution of the *PRPF31* and *TFPT* core promoters in several mammalian species might shed light on the complex regulation of these genes and the failure of mouse models of *PRPF31*-adRP. The present study design was based on the results of the study on the human genes [15], through the identification of regions homologous to the active human DNA fragments. The homologous genomic regions were tested by dual-luciferase reporter assay in order to assess conservation of gene regulation. Where homology with the human region was found to be low, novel fragments were assayed.

## Methods

### Bioinformatic analysis

Evolutionary conservation of regions was analyzed using ECR browser in NCBI DCODE software suite using default software settings for each program [17].

PAZAR transcription factor work space was used to find TFBS for *PRPF31* and *TFPT* promoters in human and monkey and bidirectional promoters of dog and mouse [18]. Transfac, Jaspar and Oreganno vertebrate profiles were used to define TFBS in conserved promoter regions. For the experimentally-defined mouse promoter region, analysis with classical vertebrate profiles of TF did not identify any TFBS.

In order to find TFBS in mouse, universal protein binding microarray data was used. UNIPROBE database (and its standard TF binding algorithm) was used to find TFBS in experimentally-defined mouse promoter sequence, using strict criterion of enrichment score =0.49 [19].

### Genomic DNA extraction

Genomic DNA was isolated from mammalian cell lines using Wizard SV Genomic DNA Purification System (Promega, UK) according to manufacturer’s instructions. Monkey DNA was extracted from cos-7 cell line, dog from MDCK cell line and mouse from IMCD3 cell line. All cell lines were purchased from ADCC.

### Fragment design and amplification

The genomic DNA sequence in the three test species was examined and fragments homologous to the three human fragments identified. In the mouse, where homology was limited, we initially looked for any conserved TFBS, but there were none observed. Therefore, fragments surrounding the *Prpf31* TSS were designed arbitrarily, according to possibility of PCR amplification in a difficult GC-rich region. In the dog, where the homologous region was approximately 2000bp upstream of *Prpf31* TSS, fragments were designed immediately adjacent to the TSS also. The twelve regions of interest were amplified by PCR using KOD polymerase (Novagen) and cloned into pGL3-basic vector (Promega, UK) in both forward (indicated by +) and reverse (indicated by −) strand orientation. Primers and PCR conditions can be seen in Table 6. In total, twelve regions were selected for assay by dual luciferase reporter assay (Figure 2).

### Dual luciferase reporter assays

The pGL3-reporter constructs were transfected into RPE-1 and HeLa cell lines. Additionally, due to concerns about species-specific transcription factor differences, mouse constructs were transfected into IMCD3 cell lines, and dog constructs into MDCK cell lines. Dual luciferase reporter assays were performed in quadruplicate, on three separate occasions. A negative control (pGL3-basic) and positive control (minimal thymidine kinase promoter, pTK) was assayed in each experiment. The transfection protocol and dual-luciferase reporter assay were performed as previously described [15]. Reporter assay data was analysed by firstly standardizing for cell number, by calculating the ratio of firefly luciferase (test) to renilla luciferase (control). This value was then compared to the pTK values, as pTK is considered a gold-standard basic promoter and, therefore, if a fragment has equivalent or greater activity, it can be considered an active regulatory region.

### *Prpf31* expression studies

Whole eye and retina tissues were obtained from DBA/2, 129S2/ Sv and C57Bl/6J adult wild-type mice, ten animals from each strain were used. The whole eye from the right side and the retina from the contralateral side were collected from each animal. Total RNA was extracted using TRIzol kit (Gibco BRL) according to the manufacturer’s instructions. cDNA was prepared using the QuantiTect^®^ Reverse Transcription kit (Qiagen) and 1 μg of RNA as template for each reaction. Real-time PCR was carried out with the GeneAmp 7500 System (Applied Biosystem). The PCR reaction was performed using 1 μl cDNA, 12.5 ml SYBR Green Master Mix (Applied Biosystem) and 400 nM primer. Water was added to make a total reaction volume of 20 μl. The PCR conditions were as follows: preheating, 50°C for 2 min and 95°C for 10 min; cycling, 40 cycles of 95°C for 15 s and 60°C for 1 min. Quantification results were expressed in terms of the cycle threshold (Ct). The Ct values were averaged for each triplicate. Both the *Gapdh* (F- GTATGACTCCACTCACGGCAAA; R- TTCCCATTCTCGGCCTTG) and *Hprt* (F- GAAGAGCTACTGTAATGATCAG; R- GCTGTACTGCTTAACCAGGG) were used as endogenous controls (reference markers). Differences between the mean Ct values of Prpf31 (F- TCGTGTGGACAGCTTCCATG; R- TTCTTCCGCTGCCCATCAAG) and those of the reference genes were calculated as ΔCt=CT*_Prpf31_*−CT*_Hprt_*_(or_ *_Gapdh_*_)_. Relative fold changes in expression levels were determined as 2−^ΔΔCt^, the *Prpf31* expression data was normalised with the DBA/2 mouse strain.

## Results

### Bioinformatic analysis

**Figure 1:**
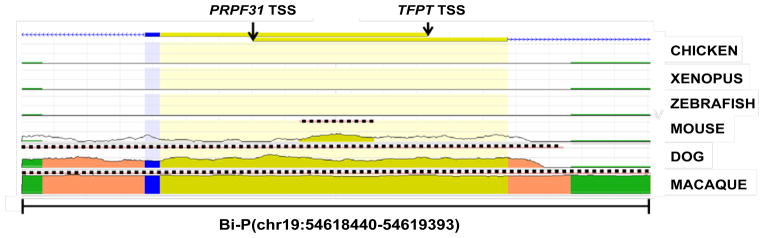
Evolutionary conservation of the Bi-P region, defined as the core promoter for *PRPF31* in the human genome. The genome architecture of PRPF31 and TFPT is illustrated, showing exon 1 and TSS of each gene. Degree of conservation is indicated by vertical height of peaks, with areas of significant evolutionary conservation highlighted by a dotted black line. Yellow – non-coding exons, blue - coding exons, salmon pink - introns, green – transposable elements and repeats.

The core promoter of the human *PRPF31* gene had previously been identified as Bi-P, the region at chr19:54618440-54619393 (hg19) and the *TFPT* promoter was contained within this region (chr19:54618440-54619133, hg19) [15]. Conservation of the defined regulatory region was analyzed in several species from different lineages, showing a remarkably low level of conservation (Figure 1). It was particularly evident that chicken, *Xenopus* and zebrafish shared no homology (defined as <25%) with the human region. In the mammalian lineage, macaque and dog shared a high level of homology (defined as >50%) with human, whereas mouse only had a low level homology over a very short distance, the majority of the defined human promoter having no homologous region in mouse. Interestingly, it was noted that although the base sequence was conserved between dog and human, the gene transcription start site (TSS) was different, meaning the homologous sequence in dog was located some 2000bp from the canine *Prpf31* TSS.

In light of these findings, three species were selected for study: *C. sabaeus, C. familiaris* and *M. musculus*. It was thought that these species would be interesting, as the green monkey had high homology to the human promoter, the dog had homology in sequence but different genome architecture and the mouse appeared to have different gene regulation entirely.

### Definition of core promoter in *C. sabaeus*

**Figure 2:**
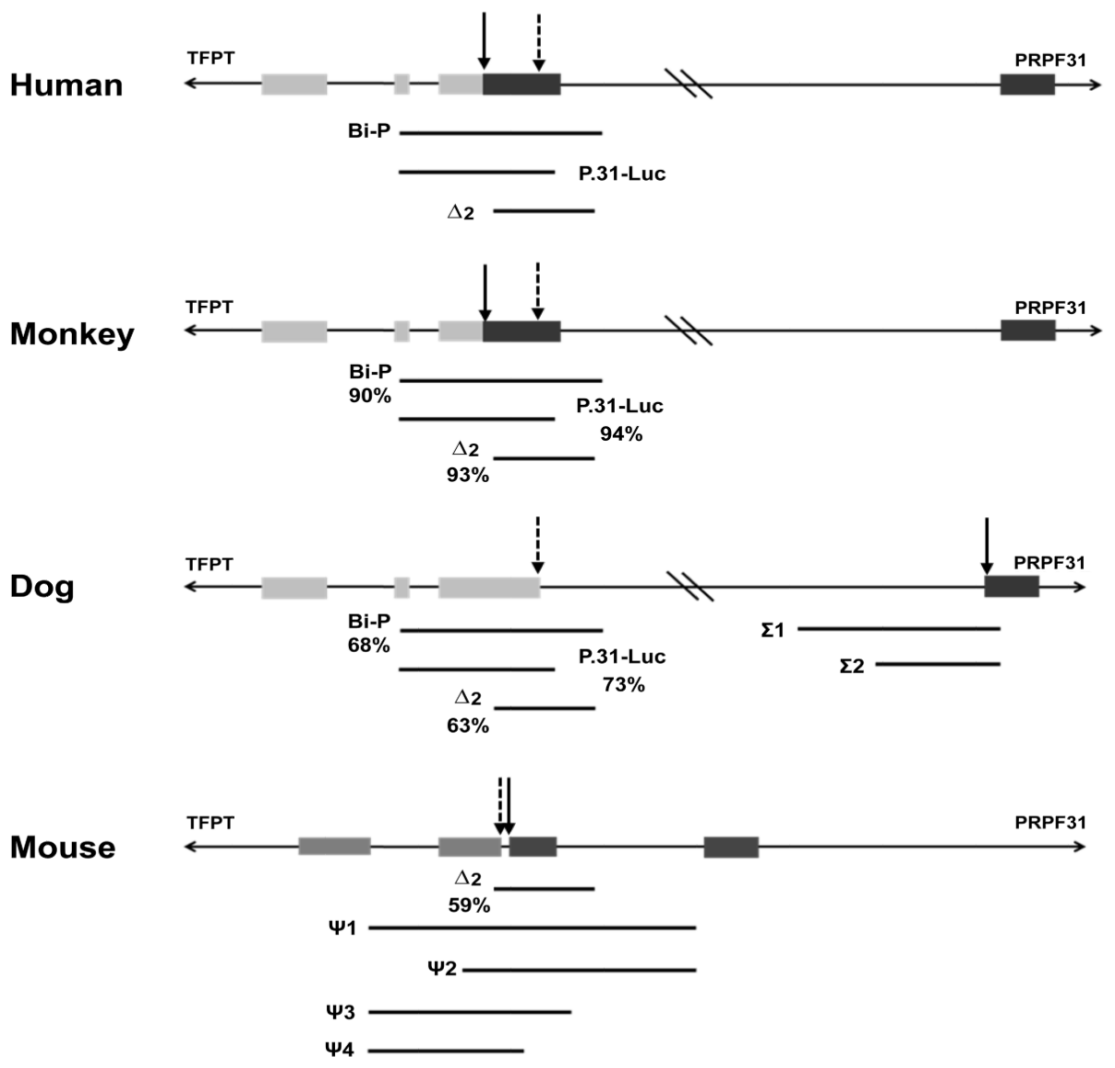
Schematic representation of the genomic regions assayed by dual-luciferase reporter assay. The three fragments with defined reporter activity in human are illustrated, as well as the homologous regions in African green monkey, dog and mouse. The *PRPF31* TSS is indicated with a solid arrow, the *TFPT* TSS with a dashed arrow. Where appropriate, the percentage homology with the human fragment is indicated.

**Figure 3:**
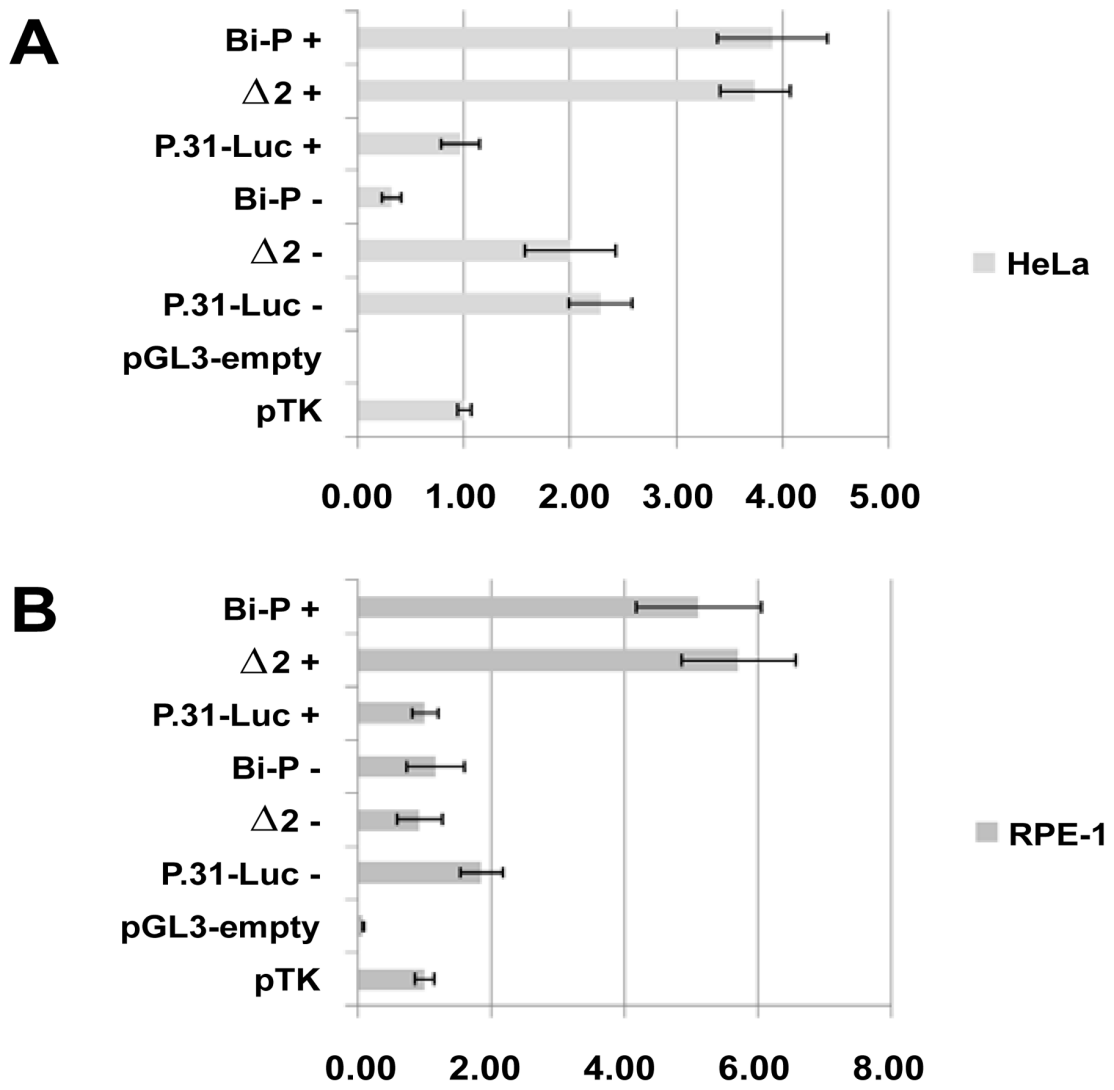
Results of dual-luciferase reporter assay using genomic sequence from *C. sabaeus* (green monkey) in HeLa cell line (A) and RPE- cell line (B). The data is presented as the average ratio of pGL3-insert to pTK, together with an error bar of ± one standard deviation, + refers to fragments tested in forward strand (*PRPF31*) orientation, − to reverse strand (*TFPT*) orientation. The absolute data values can be seen in Table 1.

Three fragments from the *C. sabaeus* (green monkey) genome that showed very high homology to the human active promoter elements were tested by dual-luciferase reporter assay (Figures 2 and 3, Table 1).

The assay showed that P.31-Luc- had the strongest reporter activity in the reverse strand (*TFPT*) orientation [2.29 ± 0.30 (HeLa); 1.85 ± 0.32 (RPE-1)], this confirmed that P.31-Luc was a core promoter with moderate activity, controlling the expression of *TFPT* in monkey. The *TFPT* promoter was, therefore, conserved between monkey and human.

In the forward strand (*PRPF31*) orientation, both Bi-P+ and Δ2+ had strong promoter activity [Bi-P+: 3.91 ± 0.52 (HeLa); 5.12 ± 0.95 (RPE-1); Δ2+: 3.74 ± 0.33 (HeLa); 5.72 ± 0.86 (RPE-1)]. It was clear that both fragments were capable of acting as strong promoter elements. There was no significant difference between the two fragments and, therefore, it was not possible to state unequivocally which was the active core promoter element *in vivo*. It is likely, however, that Bi-P is the promoter fragment, as Δ2 does not contain the gene TSS, and would not, therefore, allow correct binding of RNA polymerase II. The Bi-P+ and Δ2+ fragments both showed strong promoter activity in human and, as such, the function of these two fragments was conserved between human and green monkey.

### Definition of core promoter in *C. familiaris*

**Figure 4:**
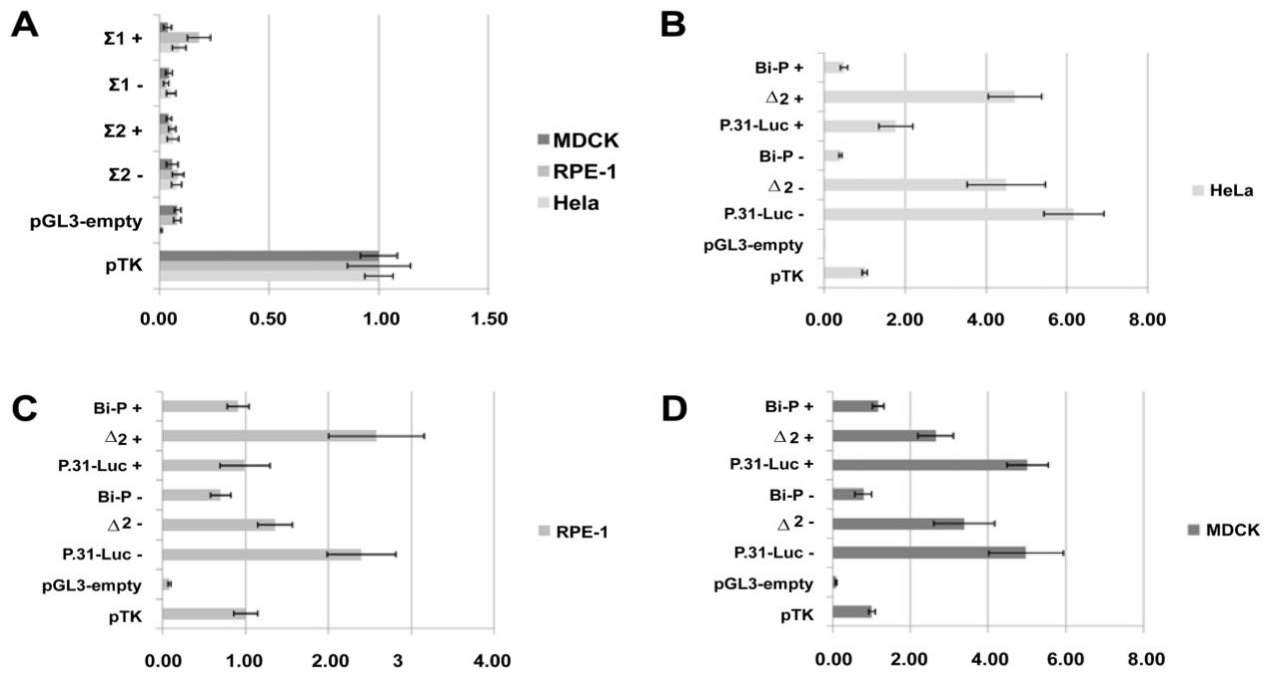
Results of dual-luciferase reporter assay using genomic sequence from *C. familiaris* (domestic dog). The region immediately upstream to the gene transcription start site were initially assayed (A), followed by regions located 2000bp upstream [(B)-Hela, (C) – RPE-1, (D) – MDCK]. The data is presented as the average ratio of pGL3-insert to pTK, together with an error bar of ± one standard deviation, + refers to fragments tested in forward strand (*PRPF31*) orientation, − to reverse strand (*TFPT*) orientation. The absolute data values can be seen in Table 2.

Initially, two fragments immediately upstream to the dog *PRPF31* TSS were designed and assayed, these fragments being homologous to intron 1 of the human gene (termed Σ1 and Σ2). Luciferase assay in HeLa, RPE-1 and MDCK cell lines showed that these fragments possessed no luciferase activity (Figures 2 and 4A, Table 2).

Subsequently, the fragments that showed homology to the human active promoter fragments – but located 2000bp from *Prpf31* TSS – were tested by dual luciferase reporter assay in RPE-1, HeLa and MDCK cell lines (Figures 2 and 4B–D, Table 2). This showed that the P.31-Luc - fragment was also the core *Tfpt* promoter in dog, indeed acting as a stronger promoter than that seen in human [6.16 ± 0.74 (HeLa); 2.40 ± 0.41 (RPE-1); 4.98 ± 0.97 (MDCK)]. The fragment homologous to the human *PRPF31* promoter, Bi-P+, did not have strong promoter activity in dog, with reporter activity less than, or very similar to, pTK [0.48 ± 0.09 (HeLa); 0.91 ± 0.13 (RPE-1); 1.16 ± 0.14 (MDCK)]. This suggests that the Bi-P+ fragment does not control the expression of *Prpf31* in the dog.

It was apparent, however, that the constituent elements of Bi-P+ (P.31-Luc + and Δ2+) had promoter activity, although this was variable between the cell lines tested. In both HeLa and RPE-1 cell lines, Δ2+ had the highest promoter activity [4.71 ± 0.67 (HeLa); 2.58 ± 0.58 (RPE-1)], whereas P.31-Luc+ showed only slight activity [1.77 ± 0.42 (HeLa); 0.99 ± 0.30 (RPE-1)]. This situation was not observed in MDCK cell line, where P.31-Luc+ possessed strong promoter activity [5.01 ± 0.52 (MDCK)], although Δ2+ also displayed good reporter activity [2.65±0.46 (MDCK)]. This suggested that the strong activation of P.31-Luc+ requires the binding of a dog-species specific transcription factor (TF), and that P.31-Luc is a true bi-directional promoter in *C. familiaris*, controlling the expression of both *Prpf31* and *Tfpt*.

### Definition of core promoter in *M. musculus*

**Figure 5:**
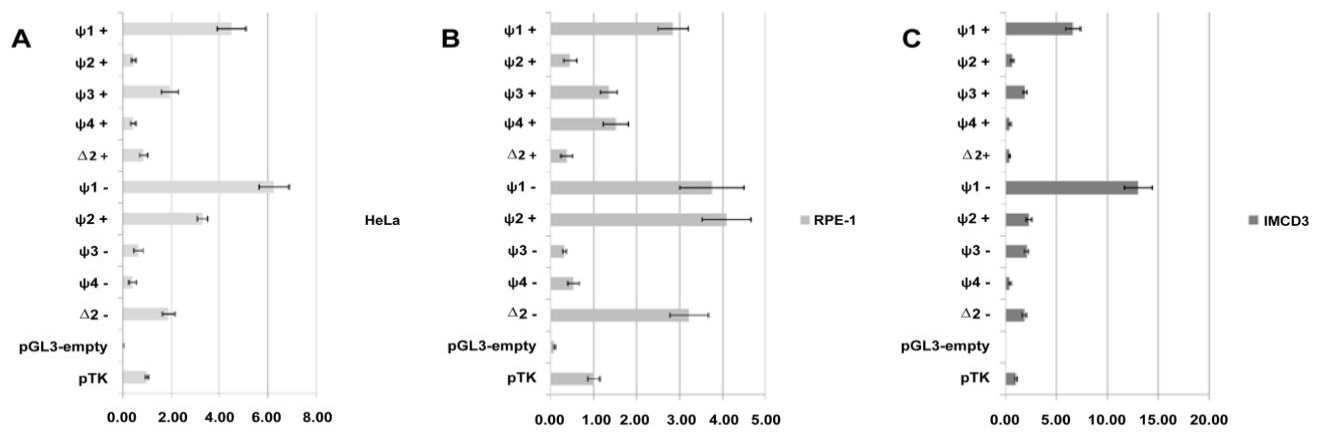
Results of dual-luciferase reporter assay using genomic sequence from *M. musculus* (house mouse) in HeLa cells (A), RPE- cells (B) and IMCD3 cells (C). The data is presented as the average ratio of pGL3-insert to pTK, together with an error bar of ± one standard deviation, + refers to fragments tested in forward strand (*PRPF31*) orientation, − to reverse strand (*TFPT*) orientation. The absolute data values can be seen in Table 3.

There was very little homology between the regions surrounding *Prpf31* TSS in the mouse genome and the corresponding region in the human genome. Of the three active human fragments (Bi-P, P.31-Luc and Δ2), only Δ2 had a homologous region in the murine genome (approximately 60% conservation). Therefore, the Δ2 fragment and four additional fragments that shared no homology with human regions (termed ψ1–4) were assayed by dual-luciferase reporter assay in both forward- and reverse-strand orientations (Figures 2 and 5, Table 3).

In mouse, ψ1 acted as a true bidirectional promoter, controlling the expression of *Tfpt* and *Prpf31*. The results were most clear in IMCD3 (murine) cell line, where ψ1 had very strong reporter activity in the forward strand orientation (6.61 ± 0.72) and the reverse strand orientation (13.02 ± 0.75). The same effect was observed in HeLa cell line (forward strand: 4.50 ± 0.60; reverse strand: 6.25 ± 0.62). In RPE-1 cell line, ψ1 had the strongest activity in the forward strand orientation (2.85 ± 0.35); in the reverse strand orientation the situation was more complex, as three fragments had relatively strong reporter activity [Δ2: (3.22 ± 0.26), ψ1: (3.76 ± 0.75), ψ2: (4.10 ± 0.22)]. This result might be due to the different transcription factor profile in RPE-1 cells, and the differences between human and murine transcription factors. Given the clear result in IMCD3 cell line (the most realistic model of the *in vivo* situation), it was concluded that ψ1 acted as a bidirectional promoter controlling the expression of both *Tfpt* and *Prpf31*.

The ψ1 region in mouse shared no significant homology with any human chromosomal region, with only very short regions (<54bp) of imperfect alignment with human chromosomes 8, 14, 19, 20 and X (Table 4).

### Prediction of transcription factor binding sites

A bioinformatic approach was taken to identify putative classical TF binding sites (TFBS) within the experimentally-defined promoters in monkey, dog and mouse. The transcription factor workspace of PAZAR was used to look for TFBS in the characterized promoters that showed conservation with human defined promoter. For mouse prediction of TFBS by pairwise conservation between mouse and dog was attempted.

**Figure 6:**
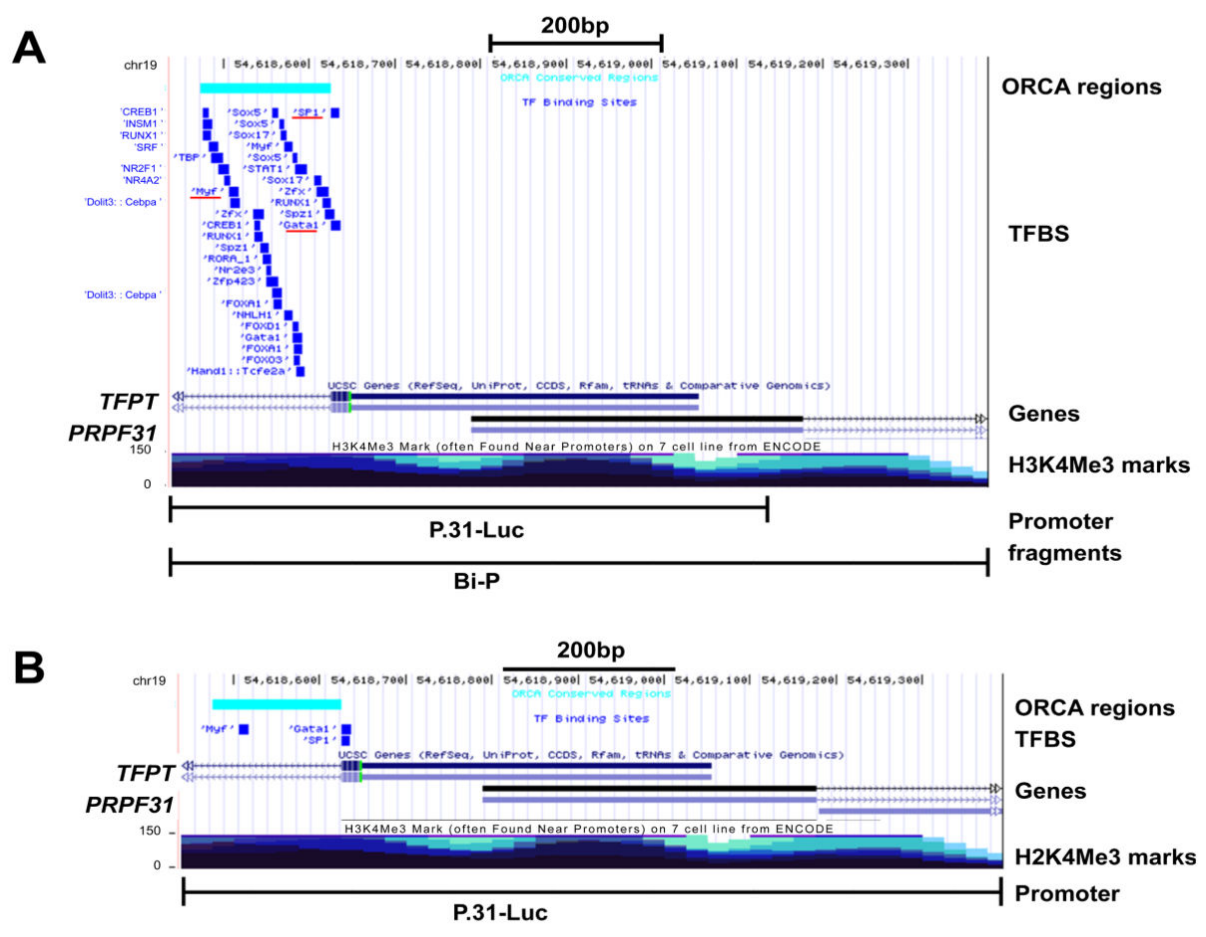
Evolutionary conserved transcription factor binding sites (TFBS) in the experimentally-defined promoters of *PRPF31* and *TFPT* in monkey (A) and dog (B). TFBS conserved between all three species (human, monkey and dog) are underlined in red on (A). ORCA conserved regions highlight areas with high level of evolutionary conservation between human and the test species. The ENCODE derived H3K4Me3 marks indicate areas that are often found in, or near, promoters.

As expected, there was a general overlap of TFBS between human and monkey, for both *PRPF31* and *TFPT* promoters (enriched in signal transduction mechanism and transcription functions) (Figure 6A). Moreover, it was observed that three TFBS (*Myf, Gata1* and *SP1*) were shared between human, monkey and dog (Figure 6A and 6B). It was not possible, however, to identify any putative classical TFBS in the mouse promoter using pairwise conservation between mouse and human or mouse and dog (scanning for standard vertebrate transcription factor profiles of Jaspar, Transfac & Oreganno). It was also observed that the human promoter region was enriched with strong H3K4Me3 mark for 7 ENCODE cell lines assayed for this histone methylation (Figure 6).

**Figure 7:**
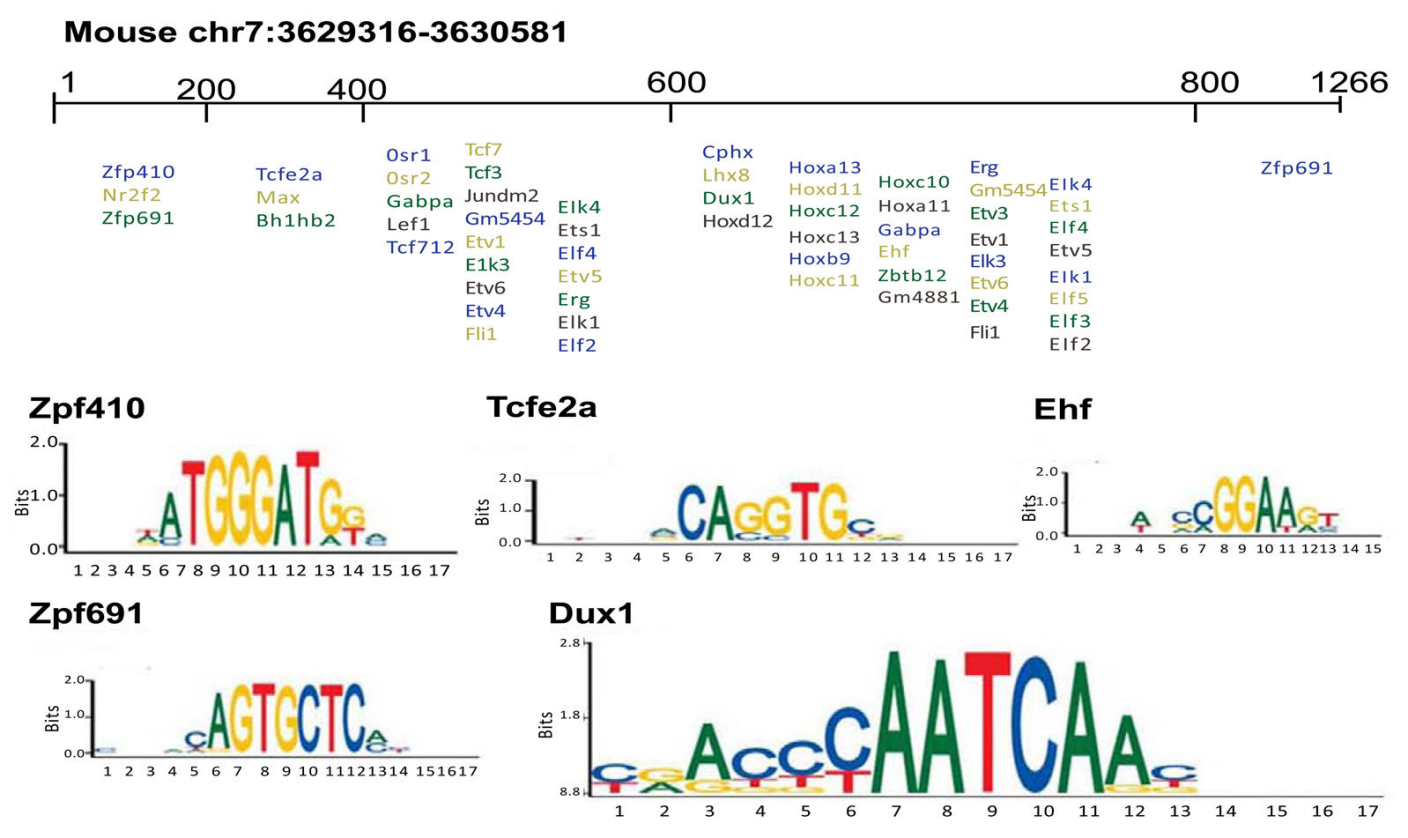
Transcription factor binding sites in the experimentally defined mouse bidirectional promoter (ψ1, located at murine chromosome 7:3629316-3630581, mm10), that were derived using protein binding microarray data, and oligonucleotide motifs of some representative transcription factors.

As a conservation based approach could not be used in mouse, due to lack of homology between mouse and human sequence, an analysis was performed using universal protein binding microarray (PBM) data, to identify putative TFBS in the experimentally-defined murine bidirectional promoter (ψ1). A strict threshold of 0.49 TF enrichment was used and all mouse PBM experiments in UNIPROBE database were searched. A range of TF having strong binding affinity to oligonucleotides of mouse promoter sequence were defined (Figure 7, Table 5). Amongst these, some TF classes were computationally predicted to bind to characterized promoter in monkey and dog (e.g. *TCFE2A*, zinc finger family, *NR2F*). However, analysis of functional enrichment showed that TFs binding to the mouse promoters were enriched in purine metabolism and also *Hox* cluster genes (which are important during development and homeostasis). These findings support the divergent evolution of promoter sequences of human and mouse, by gain of a new function in the mouse lineage.

### Study of *Prpf31* expression in *M. musculus*

**Figure 8:**
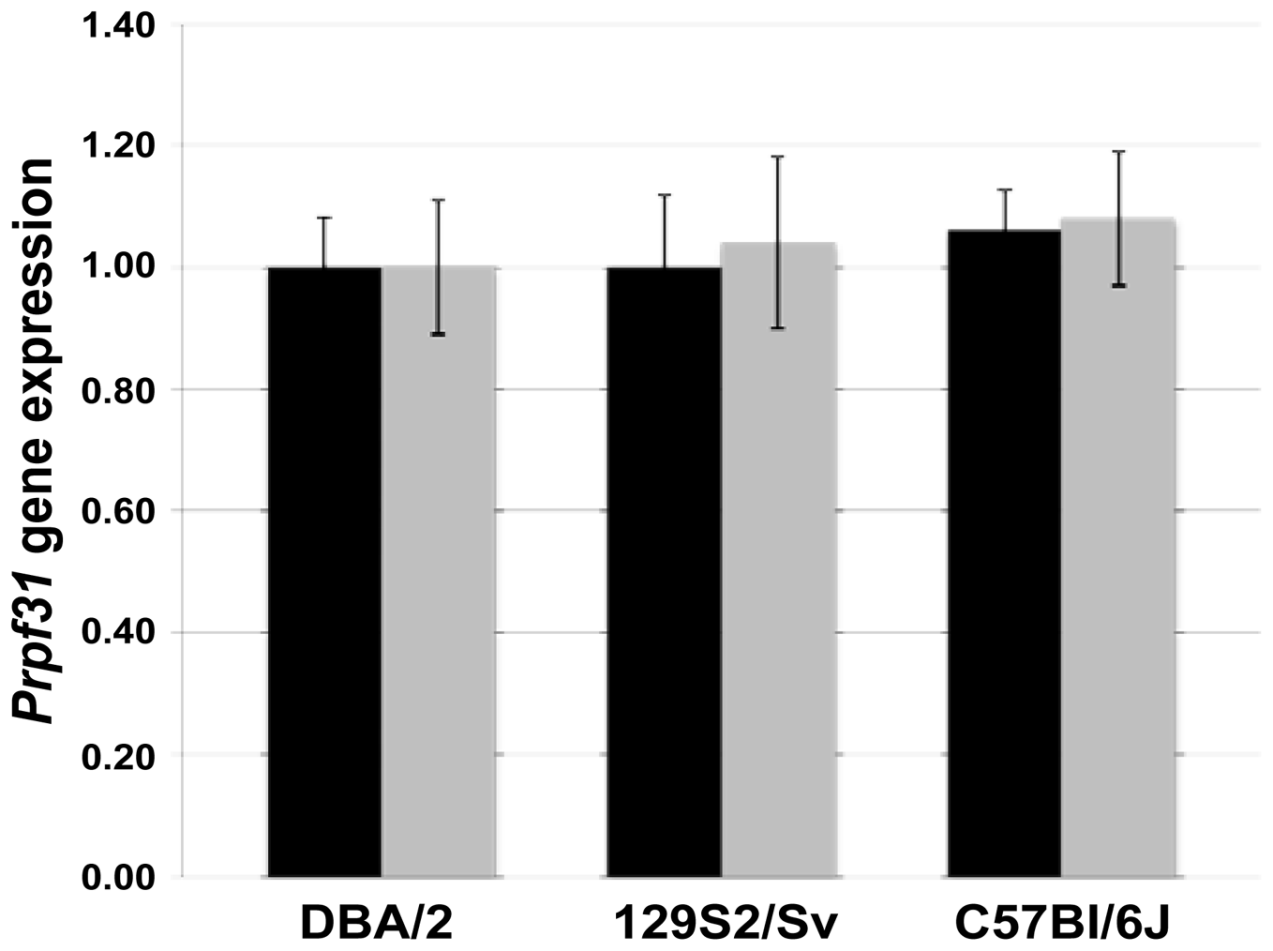
Expression of *Prpf31* in whole eye (black bars) and retina (grey bars) of three mouse strains. Data are normalized with the DBA/2 mouse strain. Error bars represent one standard error.

In human populations, *PRPF31* displays differentially expressed wild-type alleles, with highly expressed alleles providing protection against the clinical manifestation of *PRPF31*-associated adRP. We sought to analyze whether there was variable expression of *Prpf31* in *M. musculus*. Real time qPCR experiments were performed, to quantify *Prpf31* expression levels in mouse eyes and retinas. In order to avoid interference due to the genetic background or the age, animals belonging to three different wildtype mouse strains (DBA/2, 129S2/Sv and C57Bl/6J) and of the same age (8 weeks old) were analyzed. To reach a statistical significant number of tested individuals, thirty mice (ten mice for each strain) were analyzed. *Prpf31* expression level was tested in the eye and the contralateral retina of each mouse. Experiments were performed using *Hprt* and *Gapdh* genes as endogenous control. There was no statistically significant difference in *Prpf31* expression levels between the three mouse strains, either comparing eye or retinal cDNAs (Figure 8). Overall, our data suggest that in the mouse population there is no differential expression of *Prpf31* alleles.

## Discussion

The aim of this investigation was to identify and characterize the core promoters controlling the expression of *PRPF31* and *TFPT* in three species, green monkey (*C. sabaeus*), domestic dog (*C. familiaris*) and house mouse (*M. musculus*).

In green monkey, the core promoter of *TFPT* was defined as a fragment (P.31-Luc) spanning −354 to +355 relative to the *TFPT* TSS, with comparatively weak promoter activity. It was more difficult to define the *PRPF31* promoter in monkey, as both Bi-P+ and Δ2+ had strong, and very similar, promoter activity. It was, however, considered unlikely that Δ2 fragment is the true core promoter element of *PRPF31*, as it does not flank the TSS. It is apparent, however, that this fragment is capable of acting as a promoter *in vitro* and is likely, therefore, to harbour a RNA polymerase II binding site and other TFBS. As Bi-P spans the *PRPF31* TSS (−406 to +584), it is more likely that Bi-P is the *in vivo* promoter, as this would allow correct binding of the RNA polymerase II. The two defined core promoters, P.31-Luc and Bi-P, are homologous to the experimentally-defined human promoters [15]. Furthermore, there were a large number of evolutionarily conserved TFBS between human and monkey species. This was to be expected, as the green monkey fragments share >90% homology and there has, therefore, been conservation of the active promoter elements.

In dog, P.31-Luc was defined as a true bidirectional promoter, controlling the expression of both *Prpf31* and *Tfpt*. P.31-Luc spanned −510bp to +208bp relative to the *Tfpt* TSS in the dog genome, and had strong promoter activity in this orientation in the three tested cell lines. The fragment shared 73% homology with the human *TFPT* promoter, so both the sequence and the function of this region were conserved between the domestic dog and humans.

The region immediately upstream to canine *Prpf31* TSS had no reporter activity; but, rather, a long-range promoter was shown to control the expression of *Prpf31* in dog. P.31-Luc was defined as the canine core promoter, and spanned −2580 to −1857 relative to the *Prpf31* TSS (genomic co-ordinates chr1:103068699-103069421, canFam3). Long-range promoter elements bind RNA polymerase II at the TSS (in the same manner as canonical promoters), but distally bound TFs are later brought into close apposition to the gene TSS by DNA looping, this allowing activation of the RNA polymerase II complex. It is unclear why the sequence that is homologous to the primate exon 1 is not transcribed in the canine lineage.

Interestingly, the Bi-P fragment, which shared 68% homology with the human *PRPF31* core promoter did not show reporter activity. The difference between humans and dog indicates that the functional TFBS in Bi-P have been lost in dog, or that new functional sites have evolved since the divergence of the primate and canine lineages; the latter is more likely, as functional domains tend to have a positive selection pressure and are, therefore, rarely lost through evolution. Analysis of TFBS identified three binding sites that were conserved between human, monkey and dog, all of which are located within the human/ canine P.31-Luc portion, which correlates with the experimental findings.

Furthermore, it should be noted that the human CNOT3 binding site was not conserved in either monkey or dog, despite the higher level of homology between the three species. It remains to be seen whether this is reflected in less variable *PRPF31* expression levels in wild populations of these species, given that variable *CNOT3* expression was described as an important modulator of *PRPF31* expression in the human genome [19]. Another interesting finding of the TFBS prediction was the finding of a conserved NR2E3 binding site between human and monkey. NR2E3 is a transcription factor that plays an important role in developmental differentiation of the photoreceptors and, after development, is specifically expressed in post-mitotic photoreceptors [20]. As such, regulation of *PRPF31* by this factor might be part of the explanation of the retina-specific phenotype of *PRPF31* mutations.

A mouse fragment, ψ1 (chr7:3629316-3630581, mm10), was defined as a bidirectional promoter controlling the expression of both *Tfpt* and *Prpf31*. It was notable that the murine *Prpf31* 5′ region shared little homology with the human *Prpf31* 5′ region; indeed, the region that controlled expression of *Prpf31* in mouse has no homologous region in man. Bioinformatic analysis of conserved TFBS demonstrated that the mouse promoter was not regulated by any shared putative TFs with either human or dog. Instead, the mouse bidirectional promoter was enriched with TFBS for *Hox* family TFs and purine metabolism TFs, indicating significant divergent evolution in the mouse lineage.

It is necessary to speculate on the bidirectional gene architecture of the *PRPF31-TFPT* gene pair, which is conserved throughout the mammalian lineage, as well as in other vertebrate species, such as chicken, *Xenopus*, anole lizard, *Pelodiscus* turtle and even the coelacanth. The phenomenon of bidirectional gene pairs has been observed across most genomes, including yeast, nematode, fish and mammalian and it has been estimated that up to 10% of human genes exist in this divergent arrangement [21]. Bidirectional genes are controlled by a bidirectional promoter and this might allow co-transcription of the two genes, in a way similar to the prokaryotic operon. Bidirectional promoters are characterized by CpG islands, that overlap the exon 1 of both genes and this strongly suggests that the level of gene expression in bidirectional gene pairs is controlled by CpG methylation [21,22]. It is unclear why the bidirectional *TFPT-PRPF31* gene pair has arisen, given that the two genes share neither protein function nor temporal expression. *PRPF31* is a ubiquitously expressed spliceosome component, whilst *TFPT* is involved in p53-independent cellular apoptosis and is thus mainly active under conditions of cellular stress. It is difficult to imagine a shared selection pressure that might have influenced the gene architecture of these two very different genes. It should be noted, however, that during cellular stress the splicing machinery genes are down-regulated and, as such, the bidirectional gene architecture might bear relevance to the complex changes in gene expression that occur in situations of cellular stress [23].

In this work, it was also demonstrated that there is not variable expression of *Prpf31* in a population of mice from three different strains. This suggests that the differential expression of *Prpf31* has arisen after the evolutionary divergence of rodent and primate lineages. It could be inferred that the different 5′ architecture of the two genes is responsible for the lack of differential expression in the mouse. It has been demonstrated that one major factor that determines human *PRPF31* expression level is repression of transcription by binding of CNOT3 to the *PRPF31* core promoter [9]. As the CNOT3 binding site is not conserved between mouse and human, it follows that differential gene expression is not observed in mouse populations. The different gene regulation is a plausible explanation of why mouse models of human *Prpf31* mutations have failed to yield a disease phenotype [11,12]. Furthermore, other *cis-*acting factors within the *PRPF31* 5′ region that are present in human, but not in mouse, might contribute to the observed phenotypic differences.

It appears then, that in evolutionary terms, the high-expressing allele is older and, since the divergence of rodents and primate lineages, a lower-expressing allele has evolved. This raises complex evolutionary questions that will need to be addressed through systematic bioinformatic analysis of phylogenetic and genomic sequencing data. These analyses might lead to a deeper understanding of this unusual situation, whereby there appears to be rapidly-evolving control of an evolutionarily-conserved gene, with different regulatory mechanisms in relatively closely related species.

## Figures and Tables

**Table 1:** Mean dual luciferase reporter assay ratio values and standard deviation (SD) using sequence from green monkey, assayed in HeLa and RPE-1 cell lines.

	HeLa	RPE-1	HeLa (SD)	RPE-1 (SD)
pTK	1.001	1.001	0.064	0.144
pGL3-empty	0.008	0.082	0.004	0.018
P.31-Luc −	2.289	1.852	0.299	0.323
P.31-Luc +	0.966	1.012	0.176	0.202
Δ2 −	1.997	0.926	0.429	0.342
Δ2 +	3.744	5.716	0.326	0.864
Bi-P −	0.322	1.168	0.089	0.432
Bi-P +	3.907	5.118	0.522	0.947

**Table 2:** Mean dual luciferase reporter assay ratio values and standard deviation (SD) using sequence from dog, assayed in HeLa, RPE-1 and MDCK cell lines.

	HeLa	RPE-1	MDCK	HeLa (SD)	RPE-1 (SD)	MDCK (SD)
pTK	1.001	1.001	1.001	0.064	0.144	0.084
pGL3-empty	0.008	0.082	0.083	0.004	0.018	0.015
Σ2 −	0.080	0.084	0.058	0.023	0.027	0.025
Σ2 +	0.062	0.058	0.043	0.025	0.015	0.012
Σ1 −	0.053	0.031	0.044	0.020	0.010	0.016
Σ1 +	0.090	0.181	0.038	0.030	0.052	0.018
P.31-Luc −	6.164	2.399	4.979	0.743	0.414	0.967
Δ2 −	4.499	1.357	3.384	0.968	0.209	0.786
Bi-P −	0.398	0.704	0.787	0.036	0.119	0.207
P.31-Luc +	1.766	0.995	5.011	0.419	0.299	0.528
Δ2 +	4.712	2.579	2.653	0.666	0.576	0.461
Bi-P +	0.485	0.910	1.168	0.092	0.129	0.143

**Table 3:** Mean dual luciferase reporter assay ratio values and standard deviation (SD) using sequence from mouse, assayed in HeLa, RPE-1 and IMCD3 cell lines.

	HeLa	RPE-1	IMCD3	HeLa (SD)	RPE-1 (SD)	IMCD3 (SD)
pTK	1.001	1.001	1.000	0.064	0.144	0.110
pGL3-empty	0.008	0.082	0.055	0.004	0.018	0.010
Δ2 −	1.910	3.219	1.822	0.258	0.447	0.192
ψ4 −	0.403	0.523	0.396	0.147	0.134	0.098
ψ3 −	0.659	0.318	2.059	0.185	0.042	0.202
ψ2 −	3.296	4.095	2.283	0.222	0.570	0.276
ψ1 −	6.253	3.758	13.023	0.624	0.753	1.356
Δ2 +	0.875	0.372	0.395	0.161	0.136	0.065
ψ4 +	0.430	1.512	0.384	0.096	0.294	0.134
ψ3 +	1.960	1.352	1.912	0.355	0.202	0.160
ψ2 +	0.449	0.451	0.642	0.083	0.152	0.127
ψ1 +	4.495	2.849	6.609	0.596	0.347	0.719

**Table 4:** Regions in the human genome that are homologous to the experimentally defined mouse promoter, demonstrating minimal homology.

Accession	Chromosome	Max score	Total score	Query coverage	E value	Max identity
NW_003571061.1	19	42.8	42.8	4%	1	78%
NW_003571060.1	19	42.8	42.8	4%	1	78%
NW_003571059.1	19	42.8	42.8	4%	1	78%
NW_003571058.1	19	42.8	42.8	4%	1	78%
NW_003571057.1	19	42.8	42.8	4%	1	78%
NW_003571056.1	19	42.8	42.8	4%	1	78%
NW_003571055.1	19	42.8	42.8	4%	1	78%
NW_003571054.1	19	42.8	42.8	4%	1	78%
NT_011109.16	19	42.8	42.8	4%	1	78%
NW_001838498.2	19	42.8	42.8	4%	1	78%
NT_011651.17	X	41	41	3%	3.5	81%
NW_001842380.1	X	41	41	3%	3.5	81%
NT_011362.10	20	41	41	2%	3.5	84%
NW_001838666.1	20	41	41	2%	3.5	84%
NT_026437.12	14	46.4	46.4	2%	0.082	91%
NW_001838111.1	14	46.4	46.4	2%	0.082	91%
NT_167187.1	8	41	41	1%	3.5	96%
NW_001839128.2	8	41	41	1%	3.5	96%

**Table 5:** Transcription factor binding sites identified within the murine promoter, found using mouse PBM experiments in UNIPROBE database.

Gene Match	K-mer	Reverse Complement	Position	Enrichment Score
Zfp410	ACATCCCA	TGGGATGT	12	0.492169
Nr2f2	TTGACCCT	AGGGTCAA	63	0.491182
Zfp691	AGGAGCAC	GTGCTCCT	90	0.494015
Zfp691	GGAGCACC	GGTGCTCC	91	0.491563
Tcfe2a	ACCACCTG	CAGGTGGT	228	0.492541
Max	CCCACGTG	CACGTGGG	348	0.494126
Bhlhb2	CCACGTGC	GCACGTGG	349	0.493300
Max	CCACGTGC	GCACGTGG	349	0.497488
Bhlhb2	CACGTGCC	GGCACGTG	350	0.498400
Max	CACGTGCC	GGCACGTG	350	0.492097
Osr1	CCAGTAGC	GCTACTGG	479	0.491743
Osr2	CCAGTAGC	GCTACTGG	479	0.495304
Osr1	CAGTAGCT	AGCTACTG	480	0.491785
Osr2	CAGTAGCT	AGCTACTG	480	0.494346
Gabpa	GCTTCCGG	CCGGAAGC	490	0.494322
Gabpa	CTTCCGGC	GCCGGAAG	491	0.493443
Lef1	TCTTTGAT	ATCAAAGA	582	0.493387
Tcf7l2	TCTTTGAT	ATCAAAGA	582	0.493092
Tcf7	CTTTGATG	CATCAAAG	583	0.492072
Tcf3	CTTTGATG	CATCAAAG	583	0.493912
Lef1	CTTTGATG	CATCAAAG	583	0.496125
Tcf7l2	CTTTGATG	CATCAAAG	583	0.496806
Jundm2	TGATGACG	CGTCATCA	586	0.493965
Jundm2	GATGACGT	ACGTCATC	587	0.495828
Gm5454	GCTTCCGG	CCGGAAGC	490	0.495590
Etv1	GCTTCCGG	CCGGAAGC	490	0.495020
Elk3	GCTTCCGG	CCGGAAGC	490	0.495800
Etv6	GCTTCCGG	CCGGAAGC	490	0.492150
Etv4	GCTTCCGG	CCGGAAGC	490	0.491930
Fli1	GCTTCCGG	CCGGAAGC	490	0.492160
Elk4	GCTTCCGG	CCGGAAGC	490	0.491940
Ets1	GCTTCCGG	CCGGAAGC	490	0.491280
Elf4	GCTTCCGG	CCGGAAGC	490	0.493140
Etv5	GCTTCCGG	CCGGAAGC	490	0.495350
Erg	GCTTCCGG	CCGGAAGC	490	0.493290
Elk1	GCTTCCGG	CCGGAAGC	490	0.494580
Gabpa	GCTTCCGG	CCGGAAGC	490	0.492650
Elf2	GCTTCCGG	CCGGAAGC	490	0.492000
Gm5454	CTTCCGGC	GCCGGAAG	491	0.492270
Etv1	CTTCCGGC	GCCGGAAG	491	0.491880
Elk3	CTTCCGGC	GCCGGAAG	491	0.494370
Fli1	CTTCCGGC	GCCGGAAG	491	0.490020
Etv5	CTTCCGGC	GCCGGAAG	491	0.493190
Elk1	CTTCCGGC	GCCGGAAG	491	0.491590
Gabpa	CTTCCGGC	GCCGGAAG	491	0.490780
Cphx	TTTGATTG	CAATCAAA	620	0.491680
Lhx8	TTGATTGG	CCAATCAA	621	0.491150
Cphx	TTGATTGG	CCAATCAA	621	0.496010
Duxl	TTGATTGG	CCAATCAA	621	0.496350
Cphx	TGATTGGC	GCCAATCA	622	0.491310
Hoxd12	GTTTACGA	TCGTAAAC	631	0.493010
Hoxa13	GTTTACGA	TCGTAAAC	631	0.492860
Hoxd11	TTTACGAC	GTCGTAAA	632	0.497210
Hoxc12	TTTACGAC	GTCGTAAA	632	0.497690
Hoxc13	TTTACGAC	GTCGTAAA	632	0.490000
Hoxd12	TTTACGAC	GTCGTAAA	632	0.498500
Hoxb9	TTTACGAC	GTCGTAAA	632	0.494760
Hoxc11	TTTACGAC	GTCGTAAA	632	0.498230
Hoxc10	TTTACGAC	GTCGTAAA	632	0.497220
Hoxa11	TTTACGAC	GTCGTAAA	632	0.497390
Hoxc13	TTTATTAG	CTAATAAA	666	0.492680
Hoxa13	TTTATTAG	CTAATAAA	666	0.493680
Gabpa	ATTTCCGG	CCGGAAAT	640	0.495780
Ehf	ATTTCCGG	CCGGAAAT	640	0.490102
Zbtb12	GGGTTCTA	TAGAACCC	707	0.492551
Zbtb12	GGTTCTAG	CTAGAACC	708	0.498015
Zbtb12	GTTCTAGG	CCTAGAAC	709	0.497873
Gm4881	CATTTCCG	CGGAAATG	639	0.490280
Erg	CATTTCCG	CGGAAATG	639	0.490560
Gm4881	ATTTCCGG	CCGGAAAT	640	0.495580
Gm5454	ATTTCCGG	CCGGAAAT	640	0.495450
Etv3	ATTTCCGG	CCGGAAAT	640	0.490630
Etv1	ATTTCCGG	CCGGAAAT	640	0.494530
Elk3	ATTTCCGG	CCGGAAAT	640	0.493980
Etv6	ATTTCCGG	CCGGAAAT	640	0.496360
Etv4	ATTTCCGG	CCGGAAAT	640	0.493710
Fli1	ATTTCCGG	CCGGAAAT	640	0.497170
Elk4	ATTTCCGG	CCGGAAAT	640	0.495700
Ets1	ATTTCCGG	CCGGAAAT	640	0.496500
Elf4	ATTTCCGG	CCGGAAAT	640	0.495400
Etv5	ATTTCCGG	CCGGAAAT	640	0.494770
Erg	ATTTCCGG	CCGGAAAT	640	0.496890
Elk1	ATTTCCGG	CCGGAAAT	640	0.493770
Elf5	ATTTCCGG	CCGGAAAT	640	0.492260
Gabpa	ATTTCCGG	CCGGAAAT	640	0.496960
Elf3	ATTTCCGG	CCGGAAAT	640	0.493910
Elf2	ATTTCCGG	CCGGAAAT	640	0.495170
Ehf	ATTTCCGG	CCGGAAAT	640	0.493370
Zfp691	TAGTGCTC	GAGCACTA	1085	0.495547
Zfp691	AGTGCTCT	AGAGCACT	1086	0.493492

**Table 6:** Primer sequence and PCR conditions used for production of genomic fragments.

Species	Fragment	F	R	Annealing Temp	Extension Time
**Monkey**	Δ2	GGAGAATCGTTTGAACCCTGGAGAC	CCACAGCAATTCTCCGCTTAGCAG	63	10, 30
	P.31-Luc	GATGACGTCTCATGCTCGCGCC	GCAGTCCAAACCCCTAGCCT	63	14, 43
	Bi-P	GGAGAATCGTTTGAACCCTGGAGAC	GCAGTCCAAACCCCTAGCCT	63	20, 60
**Dog**	Σ1	CTCTACAGCCCACTCACCATCTTG	GCTCTTTTCCACATCATGGGAC	63	27, 82
	Σ2	CTCTACAGCCCACTCACCATCTTG	CCTAGTTCTGGGAGGTTTGTTC	63	14, 43
	Δ2	GCTCTAGCGACCTAAACCCGTCTC	CTAGGGCGATGCTCCGCTTAGCA	63	11, 33
	P.31-Luc	CCCACTGATGACGTCCCAGGC	GAACTCCAACTCCTAGCTTTTTTCC	63	14, 43
	Bi-P	GCTCTAGCGACCTAAACCCGTCTC	GAACTCCAACTCCTAGCTTTTTTCC	63	20, 60
**Mouse**	Δ2	CTTTGCCTAACCCGGCTCTT	CCAAAGCGAATCTCTGTTTAGC	60	10, 30
	ψ1	CATCAGCCAGAGACATCCCAAG	CAGACCCTAGCCTCCTCCCTC	60	25, 75
	ψ2	CATCAGCCAGAGACATCCCAAG	CCAAAGCGAATCTCTGTTTAGC	57	16, 48
	ψ3	CTTTGCCTAACCCGGCTCTT	CAGACCCTAGCCTCCTCCCTC	57	20, 60
	ψ4	GGCTCTCTTTGATGACGTTTAC	CAGACCCTAGCCTCCTCCCTC	57	14, 40
